# Perceptions of Somali American male young adults in Salt Lake City about social media marketing of e-cigarettes: a pilot study

**DOI:** 10.3389/fpubh.2026.1819564

**Published:** 2026-07-15

**Authors:** Olanrewaju Onigbogi, Kebba Kah, Rebekah Pratt, Roger Vilardaga, Kolawole Okuyemi

**Affiliations:** 1 Department of Family Medicine, Indiana University, Indianapolis, IN, United States; 2 Department of Family Medicine and Community Health, The Program of Health Disparities Research, University of Minnesota, Minneapolis, MN, United States; 3 Department of Implementation Science, Wake Forest University School of Medicine, Winston-Salem, NC, United States

**Keywords:** e-cigarettes, perceptions, Somali American, targeted marketing, young adults

## Abstract

**Background:**

Targeted marketing of tobacco products affects immigrant youth in the United States. Somali Americans may be more vulnerable because of the high prevalence of smoking among the population. This study therefore sought to determine the perceptions of male young adult Somali Americans in Salt Lake City about targeted social media marketing of e-cigarettes.

**Methods:**

We conducted three focus group sessions in May 2023 for males aged 21–25 years who self-identified as Somali Americans and had lived in Salt-Lake City for at least 6 months. A focus group guide was developed to explore the experiences and perceptions of social media marketing of e-cigarettes, and a deductive thematic analysis of the data was used to identify, analyze, and report patterns.

**Results:**

There were twenty-four participants in three focus groups. Five main themes were identified, namely: 1) intrusiveness in relation to perceived access by third parties to social media history related to e-cigarettes; 2) vulnerability, including the tendency to feel overwhelmed by a lack of control over social media communication; 3) anxiety about the possibility of social exclusion due to e-cigarettes; 4) enjoyment of the glamor associated with e-cigarette product images and interaction with social media influencers: and 5) pro-active creativity which addresses mental activities done to truncate the flow of e-cigarette advertisement.

**Conclusion:**

The feeling of intrusiveness, vulnerability and increased anxiety among the participants are indicative of negative perception of their experiences. Although the feelings of enjoyment can potentially result in lowered risk perception of e-cigarettes, there is a need to further explore the experiences of proactive creativity among participants as it can be used in designing effective interventions to reduce e-cigarette use in this population.

## 1 Introduction

Electronic cigarettes (e-cigarettes) are the most used nicotine/tobacco products among young adults (aged 18–24 years) in the United States (1). According to data from the Population Assessment of Tobacco and Health (PATH) conducted in Year 2020, a total of 14.5% of adults aged 18 to 24 reported regular use of e-cigarettes (2). Data from the National Center for Health Statistics (NCHS) 2021 estimated current e-cigarette use among young adults aged 18–24 to be 11.0% (11.6% among men and 10.3% among women) (3). Moreover, there have been mixed outcomes with the use of e-cigarettes as a medium for reducing the harmful effect of combustible cigarettes with recent reports indicating that e-cigarettes may in fact contribute to continuing nicotine dependence (4–10).

Research shows that promotion of health behaviors such as smoking can disseminate rapidly on social media through exposure to images or depictions of tobacco use (11). Unlike advertisements of combustible tobacco products such as cigarettes, which is banned, the promotion and advertisement of e-cigarettes or Electronic Delivery Systems (ENDS) on social media is permitted if they include a health warning statement (12). These requirements established and enforced by the US Food and Drug Administration went into effect in year 2018 (13). Vaping companies often contract social media influencers, who are paid for their ability to target a niche audience of potential buyers, particularly on social media. This type of promotion is difficult to regulate because it is guided by the Federal Trade Commission, whose only guideline for the activity is for influencers to disclose relationships between them and brands (13). This regulatory gap has been exploited by some social media influencers to promote vaping, with recent studies highlighting low compliance with regulations across board (14, 15).

Past studies have reported targeted marketing of combustible tobacco products to marginalized communities by multinational tobacco companies (16–18). Suspected activities range from disparities in sighting of tobacco advertisement billboards (19–22), targeted marketing of mentholated cigarettes to Black people (19) and marketing of “slim cigarettes” to women (23). In addition, there have been reports of racial disparities in total tobacco marketing volumes with higher rewards per volumes of sales deliberately set to incentivize retail marketers in marginalized neighborhoods (16, 22, 24). Tobacco advertising and promotion activities have been shown to correlate with high smoking rates in Black communities (25–27). In addition, youth and young adults report greater brand recognition of tobacco products compared to their white counterparts, and Black young adults report a link between their use of e-cigarettes and previous exposure to at least one appealing advertisement (28–32). Black adolescents who are exposed to higher numbers of tobacco outlets and advertisements have also been shown to demonstrate greater intention to smoke in the future and are more likely to report cigarette purchase (25, 28).

Social media use is known to result in a digital footprint, which refers to the data trail of online activities such as online purchases, social media interactions and browsing history (33, 34). Tobacco product manufacturers have been accused of utilizing this information to target people particularly on popular social media platforms with a focus on demographic groups such as youth and racial/ethnic minorities by leveraging on their online behaviors and cultural preferences (35–37). Research has also shown that psychological characteristics of users can accurately predict their digital footprints, such as their Facebook Likes or Tweets (14, 38).

Certain psychological effects of targeted marketing such as negative self-perception and unrealistic expectations fueling feelings of inadequacy, anxiety, and low self-esteem have been reported in previous studies involving vulnerable populations (39, 40). Other effects that have been reported include reduced risk perceptions and stimulation of curiosity (41). A closer look at these effects is important because the current practice may lead to exploitation of personal data to foster commercial gain, reinforce social biases, cause emotional distress and target the vulnerable. In addition, targeted advertisements can reinforce existing beliefs and limit exposure to diverse perspectives, hindering critical thinking (39, 40). It may also worsen feelings of envy, anxiety, and depression and reinforce harmful stereotypes, impact users' mental health and perpetuating negative self-perceptions, especially in marginalized groups such as Somali Americans with an estimated adult male smoking prevalence of 44% which is disproportionately higher than 14% prevalence in the general U.S adult population (42, 43). In addition, Somali immigrant youth have been reported to experience significant family and social disruptions which may affect their perceptions of targeted marketing of e-cigarettes and make them more vulnerable to addiction (33, 34). This pilot study therefore sought to determine the perceptions of Somali American male young adults in Salt Lake City, Utah about social media marketing of e-cigarettes.

## 2 Materials and methods

### 2.1 Overview

We conducted three focus group discussion (FGD) sessions among Somali American males aged 21–25 years in May 2023. Focus groups were chosen to elicit both shared and divergent themes expressed by the participants and facilitate in-depth conversation on experiences and perspectives about e-cigarette marketing and use.

### 2.2 Theoretical framework

We took a post-positivist and constructivist approach with our research questions and employed a deductive template analysis approach to collecting and interpreting our data (44). The *a priori* themes were derived from studies that had examined the health effects of social media targeted marketing including participant experiences, views and attitudes (33–36). The Kessler (K6) psychological distress scale distress scale was informative in designing focus group questions exploring perceptions of distress among participants. The scale was used to guide the questions because it assesses items such as anxiety, sadness, hopelessness, mental exhaustion, restlessness and worthlessness and has been validated in other studies (45). We did not seek to compute a score based on the perceptions of participants but designed the questions to cover key concepts of psychological distress.

### 2.3 Study setting

Focus group sessions were conducted in community locations, which provided easy access and privacy for participants. The sessions were conducted in person with the venue provided by the local contact organization. Each focus group took about 60 min, was conducted in English language and audio recorded.

### 2.4 Procedures

Facilitators used a semi-structured focus group guide which was developed by the study team through literature review and discussions with subject matter experts (Supplementary Table 1). The guide comprised of questions exploring the e-cigarette and social media experiences of participants. There were also questions on social media marketing of e-cigarettes and participants' perceptions of psychological distress. Sessions were transcribed in preparation for analysis and coded using the Dedoose software. The study was reviewed and approved by the University of Utah Institutional Review Board (IRB) number 00159355 on 30th September 2022. Information sheets were given to participants by the team members who commenced the group discussions after the verbal assent of the participants.

### 2.5 Participants

Participants were selected using purposive sampling techniques. Recruitment efforts were facilitated by our partner non-governmental organization with regular outreaches to the Somali community in Salt Lake City. Participants received a $50 gift card as compensation for their time in accordance with the approval obtained from the IRB with an informed consent document signed by individual participants after they were introduced to the study by the Principal Investigator (PI).

We purposively recruited only Somali American males because previous studies indicate low rates of tobacco use among females (42, 43). Other criteria included self-identification as being of Somali origin, ability to communicate in English at least to the 8th Grade level by the Flesch-Kincaid Grade classification and residence in Salt Lake City for 6 months or longer. Previous vaping or smoking experience was not a criterion for recruitment. Furthermore, our study was restricted to young males between 21 and 25 years of age because we could not obtain the IRB permission to conduct FGDs among persons less than 21 years of age as they were not legally permitted by Utah anti-tobacco laws to be directly exposed to any tobacco-related information.

### 2.6 Data analysis and interpretation

Thematic analysis, which provided a structured process for summarizing data and flexibility to recognize emergent themes and interpreting meaning was used to analyze the data (46). The team identified recurring narratives, and insights into the psychological effects of targeted marketing of e-cigarettes within this specific population in line with *a priori* themes which had been derived from previous studies. This activity required iterative discussions among investigators during the development of coding structures and interpretation of meaning. We thereafter developed a coding structure and identified a variety of sub-codes. The major themes and main ideas that recurred across each focus group and quotes that encapsulated themes and trends were then highlighted by the two researchers who independently conducted the coding exercise (Supplementary Table 2). The two coders achieved an 85% overall raw percentage agreement across all coded transcripts and intercoder disagreements were resolved after they came to a consensus at review meetings. The coders reviewed the disputed data segments by referring to the focus group transcripts, stated their rationales based on the codebook definitions, and debated until they reached a mutually agreed-upon code. There was no need for third-party adjudication in any of the disputed cases.

### 2.7 Research team and reflexivity

Co-authors OO and KK recruited and consented all participants and conducted the three focus groups. The PI, OO, was undergoing the health equity research post-doctoral fellowship at the University of Utah at the time of the project and KK, who worked as the note-taker and timekeeper was a PhD student. Both facilitators were African immigrants themselves and were able to interact with participants in this respect. Both being male, they were also best matched to a study involving male Somali. Within the context of the current study, both researchers who were involved in face-to-face contact with study participants took care to ensure that they did not unduly influence them based on their own background, experiences and prior assumptions. The researchers deliberately adopted a facilitation approach in setting the scene for the interviews. The interviews took place in venues that participants were familiar with to minimize any feeling of being under duress.

## 3 Results

A total of 24 young adults participated in the study with an average age (M = 22.9, SD = 2.1) years out of which 16 (66.6%) had completed at least high school education. Five major themes emerged from our analysis of the focus group sessions: 1) Intrusiveness; 2). Vulnerability; 3) Anxiety; 4) Enjoyment 5). Pro-active creativity. The main themes along with illustrative quotes are outlined below. Additional quotes can be found in Supplementary Table 2.

### 3.1 Intrusiveness

Participants perceived that there was an intrusive link between their internet use and social media history, e-cigarette messages and suggested videos and reels. They shared that they lacked an understanding of the algorithms guiding content delivery to them on social media and therefore felt that their sense of privacy had been violated. Participants also voiced views about their perception of being monitored online with some of them sharing specific experiences with intrusion in which real-time verbal discussions with others about e-cigarettes resulted in the flow of “nudges” of e-cigarette advertisement materials on social media. They felt that social media algorithms related to e-cigarette advertisements were designed to maximize engagement and prioritize content that falsely reflected their preferences. Most participants had the opinion that there was opacity in algorithm design which they thought made them more vulnerable to targeted marketing of e-cigarettes and associated paraphernalia.

“*I receive e-cigarette messages whenever I chat with others about pods.” (Participant in FGD1)*

“*I've learned not to view vaping messages. When you do, they keep coming.” (Participant in FGD 1)*

“*I have bought vaping products online in the past. Now I get both e-cigarette and vaping cessation messages.” (Participant in FGD 3)*

“*I recall searching for some vaping products out of curiosity in the past but have continued to get e-cigarette promotion prompts.” (Participant in FGD 2)*

Participants described that the sense of intrusiveness could amplify previous traumatic experiences related to tobacco use. Participants expressed that they could not help but relate their current sense of intrusiveness to the guilt they felt when they were caught using tobacco at an earlier age. They reported that the e-cigarette advertisements reminded them of the discomforting feeling which had continued to haunt them as they replayed the script in their minds. They reported that their trauma was further amplified when other young people saw e-cigarette advertisements on their mobile phones even if the people neither expressed surprise nor made any negative statements related to their observation.

“*Every time I see an e-cigarette advert, I feel guilty because I used to vape in middle school.” (Participant in FGD 2)*

“*These vaping messages remind me of a bad experience I had when I was caught smoking in Middle School. The shame was overwhelming.” (Participant in FGD 2)*

“*In Islam, cigarettes are impure substances which should never be put in the mouth. I feel embarrassed when my Muslim friends see vaping advertisements on my phone because they know that I used to smoke.” (Participant in FGD 3)*

In addition, some participants shared that they felt there was some manipulation of their individual social media pages which made them the preferred targets of e-cigarette content. In sharing their past experiences, some participants described past events in which their social media accounts had received unapparent e-cigarette content of cheap vape pens which resembled standard ink pens, styluses, and highlighters. They also described how easy it was for them to click the advertisements which led then to sites with disposable e-cigarette paraphernalia. In addition, they described how they thereafter received e-cigarette advertisements repeated over a number of days. They shared their opinion that they thought it was incorrect for advertisers to hide their true intentions and “click bait” them into visiting the e-cigarette sites.

“*I have received many messages that I did not know were related to vaping until I clicked the link.” (Participant in FGD 2)*

“*I get prompted to view pens without knowing that they are not writing pens.” (Participant in FGD 2)*

Participants also shared that all the social media influencers who had tried to reach out to them with e-cigarette content were young and of African origin. They said they had also received messages with pictures showing vape pods with colors of common fruits. They felt that the messaging may have been targeted at them based on their profiles on social media.

“*I get a lot of e-cigarette content from young, and Black influencers.” (Participant in FGD 2)*

“*I get pictures of pods that look like common fruits.” (Participant in FGD 1)*

### 3.2 Vulnerability

Participants expressed the opinion that exposure to e-cigarette advertisements on social media promoted negative experiences about their lives and personal appearances. They shared that they felt vulnerable almost every time they got these advertisements and that this feeling was not assuaged by their realization that the images may have been manipulated. They also expressed the opinion that this experience may be related to their personal struggles with insecurity and the desire for continuous validation among peers. Some participants felt that they were particularly vulnerable to social media influencers to whom they could relate with by virtue of age, race or cultural background. They also shared that they felt vulnerable at the sight of expensive clothing and ambience which influencers always had around them. They described that the constant internal tension of comparing their everyday life with these fictionalized ideals resulted in decreased self-esteem. In addition, participants reported that e-cigarette promotion on social media usually came with images of success, with the accompanying glamor, creating unrealistic expectations about body image, success, and happiness. The effect of these unrealistic images was emphasized in greater detail by participants who reported that they were not born in the US and that the photographs and videos made by e-cigarette promotion models negatively impacted their self-esteem. The effect was highlighted by few respondents who were in the final year of their tertiary education journey and felt that they were under some pressure to be perceived as successful.

“*All the messages I get are from well-dressed people my age and I always feel like I need to do more about my dressing.” (Participant in FGD 1)*

“*The background of vaping pictures always looks colorful and cool. They usually have a pen in one hand and a symbol of success, for example, a good-looking car on the other.” (Participant in FGD 1)*

“*The people in the videos look happy and remind me of the need to be successful.” (Participant in FGD 2)*

Some participants also shared the frustration with their inability to keep social media engagement focused on religious content which was their primary interest. They reported that they always ended up getting unwanted content which they could not evade despite best efforts. Participants voiced the opinion that they sometimes felt like they had few options apart from closing such accounts.

“*I'll continue to indicate my lack of interest in e-cigarette materials. I am interested in religious content and look forward to a time when only religious content will get to me.” (Participant in FGD 3)*

“*I create new accounts and then stop using them after some time. I feel like there is very little choice that I have about content.” (Participant in FGD 2)*

### 3.3 Anxiety

In general, participants reported some anxiety arising from uncertainty about outcomes of interactions. They also reported that they sometimes felt overwhelmed by “pop up” e-cigarette advertisements on their devices and were pessimistic about the outcome of engaging with the social media companies on the subject. The sense of anxiety expressed by the participants was also related to the possibility of getting messages on other devices that were linked to their social media accounts. Some participants shared that they had received e-cigarette advertisement and promotions on their email accounts which they feared may expose them to ridicule among professional peers.

“*I report vaping advertisement that ‘pop up' on my page. However, the messages continue for such time that I believe I need to do more with my life.” (Participant in FGD 1)*

“*The vaping advertisements on my phone come up at the wrong time.” (Participant in FGD 3)*

“*Vaping advertisements do not only appear on my phone. They appear on all devices linked to me including my email.” (Participant in FGD 3)*

Moreover, a feeling of anxiety was commonly shared among the participants, especially when they reported attempts to engage social media companies in reducing the volume of e-cigarette messaging on their social media accounts. They shared some feelings of inadequacy due to their inability to predict outcomes after they identified problems and tried to engage social media companies. They also felt that they did not have enough channels through which they could air their grievances against the social media companies, when they perceived that they were being targeted with e-cigarette messages. There was consensus on the opinion that social media companies were unduly favored in the power equation when it came to messages on their professional and social accounts.

“*Social media companies make much money from the adverts. I don't think they are interested in the effects of the e-cigarette advertisements.” (Participant in FGD 3)*

“*I immediately report unwanted e-cigarette advertisement using the built-in help center in Instagram. However, it takes a lot of time before it goes away. I don't know what else that I can do to make it go away faster.” (Participant in FGD2)*

In addition, participants expressed anxiety about the risk of a fall in social ranking among their Somali female peers if they were associated with e-cigarette use. Some of the anxiety related to navigating cultural differences in how their friends of a different sex in the larger American society viewed vaping, and how that differed from how Somali young females viewed the practice. Participants expressed the opinion that due to the cultural and experiential backgrounds of their Muslim peers, being linked with e-cigarette use could attract a negative label.

“*I always fear that my female friends will see the e-cigarette adverts and would be disappointed in me.” (Participant in FGD 1)*

“*I feel uncomfortable anytime I open my browser when my female friends are nearby.” (Participant in FGD 1)*

### 3.4 Enjoyment

Participants shared exciting experiences of watching e-cigarette content and receiving personalized messages and exchanges with social media models whom they had always looked up to. They also expressed the sense of fulfillment and joy that had accompanied their interaction with models whom they looked up to on the social ladder. Some participants shared their views about the feelings of enjoyment when viewing images of success and beauty associated with the e-cigarette promotions. They also reported that despite their knowledge that the images may not be real, they loved them because they were always shot in a good ambiance and the models looked attractive. The respondents also confessed that they enjoyed viewing e-cigarette advertisements despite being aware of the risks involved and described the contrasting emotions of enjoying curated content.

“*I feel like I'm not prepared for more social media use. I use little social media these days. There is so much to enjoy looking at the nice clothes and good-looking people.” (Participant in FGD 2)*

“*I agree with people who say that there is more to social media than what we see. However, I like what I see, and I enjoy watching the people.” (Participant in FGD 3)*

Some participants also shared that they enjoyed interacting online with the social media influencers who marketed e-cigarette products and that due to the quality of the interaction, they could not help but perceive them as being down-to-earth, trustworthy and knowledgeable. They reported interaction which varied from the exchange of promotional materials to information sharing about educational sources related to e-cigarettes. In addition, some participants reported that the casual display of vapes and other paraphernalia by these social media influencers made it easy for them to identify vaping paraphernalia even though they had never seen the products in real life.

“*I enjoy interacting and following the influencers (e-cigarette influencers) on social media because they are the leaders and I could benefit if they follow me back.” (Participant in FGD 1)*

“*The influencers who talk about vaping are very approachable and real. They look happy and remind me of the need to be successful.” (Participant in FGD 2)*

“*I see the things (paraphernalia) used to smoke e-cigarettes every time online. I can easily recognize them when I see them with young people.” (Participant in FGD 2)*

### 3.5 Pro-active creativity

Pro-active creativity refers to deliberate human-led creative efforts to actively disrupt how social media algorithms perceived the participants. The creative activities were designed to break the automated feedback loops which they thought were designed to serve specific advertising platforms. Participants shared their views about success recorded in efforts to reduce the flow of e-cigarette advertisements to their social media pages. Some participants expressed their excitement about the forced creativity at the individual level. Most of the efforts that they spoke about revolved around conducting personal research and identification of the need to keep away from using items with personal identifiers such as bank cards and drivers' licenses in e-cigarette transactions. They also reported the innovation of using only public computers in the search and purchase of e-cigarette-related items.

“*I have had some peace since I adjusted my social media privacy settings which makes it difficult for marketers to target my account.” (Participant in FGD 1)*

“*I have learnt to only buy (e-cigarette) cartridges and pods with cash in the shops and gas stations.” (Participant in FGD 2)*

“*I deliberately delete tracking identifiers on my computer to block feedback to advertising (e-cigarette advertising) sites. I have found that this helps me a lot.” (Participant in FGD 2)*

Participants expressed that they had been forced to become creative in the manner of approach for communicating information on possible targeted marketing of e-cigarettes with their Somali American peers. They reported that in order to avoid potential psychological distress that may be associated with initiating the subject of smoking or vaping, they had found it easier to share generic information about how to prevent targeted marketing from online vendors in general. Some respondents expressed satisfaction with the extent to which local transfer of knowledge on how best to prevent-e-cigarette marketing on social media had been of help to them.

“*When I see e-cigarette adverts on my friends' phones, I muster the courage to open the conversation telling them to look for a report link or button on their App.” (Participant in FGD 2)*

“*I know that e-cigarette targeted messages wouldn't stop until we do something about it. I have shared information with my friends about how they can get the messages to stop.” (Participant in FGD 1)*

## 4 Discussion

Several studies have established the link between tobacco advertising and adolescent smoking behaviors for both combustible and non-combustible tobacco (47, 48). Our study provides a unique perspective because it focuses on the perceptions of a group of young adults on the psychological effects of targeted marketing of e-cigarettes, a product which has been marketed as a safer alternative to combustible tobacco (49, 50). Our study is also unique because it highlights the perspectives of targeted marketing of e-cigarettes during a phase in life (young adulthood) in which studies have identified high levels of vulnerability (51). In addition, this study sought to better understand the perceptions on the psychological aspects of e-cigarette advertisement and its effect on male Somali American young adults. To our best knowledge, this is the first time that this specific type of study has been conducted in this population. This finding is important because this population has grown in the US by more than three-fold over the past few decades with the potential for further growth due to high birth rates and the maturation of the U.S.-born second generation (52, 53).

Somali American adolescent and young adult immigrants to the U.S, like many of their peers from other countries have been reported to be particularly vulnerable to addictions and other mental health challenges probably due to past traumatic experiences (42, 43, 53). Studies have also shown that trauma is an embodied experience that remains encoded in the nervous system and physical sensations, somatic and can be transferred through generations (53–55). Therefore, it is important that this population which may have experienced past psychological, emotional and cognitive trauma are shielded from future possible stress due to targeted marketing practices. Our results also highlight that the feeling of intrusiveness and anxiety among the participants include trauma and shame associated with tobacco use misadventure in adolescence or at an earlier age. This finding is important because it shows that despite the high adult male smoking rates, underage tobacco use in this population still appears to attract social sanctions. This finding aligns with nationally available US data which failed to identify high adolescent smoking rates in this population (1, 2). Our findings on the fear of negative perceptions of the female peers reported by the participants also aligns with reports of Somali female smoking rates that are a lot less than the US national average (1, 2). Future studies conducted to determine the barriers and facilitators of this trend and the average age of smoking initiation in this population will help to design interventions which could help delay the average age of smoking initiation in the US general female population.

Studies have shown that social media influencers are pivotal in shaping consumer-brand relationships, increasing advertisement engagement and reducing product harm perceptions (56, 57). Our study participants cited the sense of personal relevance and satisfaction that came from interacting with social media models and influencers whom they looked up to on the social ladder. In this way, our results align with findings from another study conducted in a similar age group (58). Previous studies have demonstrated a mix of emotions among youth who are primarily drawn to social media for happiness as an instant gratification but whose emotion gets negated by the psychological stress accompanying social comparison (59, 60). Our study also highlights the tendency for young adults to measure their self-worth in comparison with peers leading to a phenomenon called “social media envy,” which is accompanied by feelings of inadequacy, depression, and anxiety (61, 62). Social media envy has been defined as feelings triggered by curated posts regarding lifestyles, appearances, or professional achievements, leading to comparison-induced anxiety. The emotions perceived by our participants appear to be a mixture of lifestyle envy which leads to the fear of missing out and appearance-related envy which is triggered by fitness or beauty content leading to unhealthy comparisons (63, 64).

The findings of our study align with similar ones conducted to examine social media interaction among youth which indicate that algorithms designed to maximize engagement prioritize contents that elicit emotional responses with by selectively choosing and presenting the best content leading to constant comparisons with idealized representations of others' lives (64, 65). In addition, we observed that our participants felt that their personal spaces were being intruded based on algorithms which they had no control over, an assumption which previous studies have also highlighted (64, 65). Furthermore, our study highlights psychologically “positive” effects from the perspective of participants. These include the enjoyment which they derived from the social media images and interaction leading to reduced perceived harm, increased curiosity, and feelings of social inclusion. These observations have been reported by previous studies (37, 66). These experiences tend to shift attitudes of potential e-cigarette users by positioning vaping as a trendy, healthy alternative to smoking, though research often highlights the negative, addictive implications of these perceived benefits (66).

Finally, our work highlights proactive creativity which is a result of the need to stem the flow of e-cigarette content to individual accounts and the innovative means through which participants shared their knowledge with peers. Although participants reported that they had encountered some psychological distress in exploring and sharing information on stemming e-cigarette advertisement, there appears to have been some overall positive effect. A combination of proactive creativity and innovation in other studies has been found to be beneficial to the overall mental health of young adults (67, 68).

### 4.1 Limitations

This study has several limitations. The small sample size of this pilot study limits reaching saturation of themes with the possibility of developing an incomplete theoretical framework on which future studies can be built. Future researchers need to be wary of the incompleteness of the understanding of the topic to forestall skewed interpretation of subsequent findings. In addition, this study considered the perceptions of respondents to e-cigarette advertisements on social media but not the products themselves. Future studies would benefit from a design that identifies specific commercial products so that recommendations about their marketing could be more specific. Furthermore, our study, which is self-reported with no external validation is limited by participants' recall bias because they may not accurately remember their experiences or may choose to answer in socially acceptable ways. We also note that our study did not seek to explore the racial identification of actors and advertisement materials which were used. This could be the subject of future research which may help to guide future policy concerning cultural affiliations of e-cigarette social media influencers. Another limitation to our study is the fact that it does not represent all Somali Americans and may not be generalizable to other immigrant groups in the US. Finally, the use of a qualitative design in determining participants' perception of the psychological effects may have been a limitation. Future designs could utilize a mixed method design with a quantitative component comprising multi-method assessment (e.g., behavioral observation, performance-based tests) using multiple scales.

## 5 Conclusions

In conclusion, our study indicates that Somali American young adults perceived that targeted marketing of e-cigarettes via social media led to feelings of intrusiveness, vulnerability and increased anxiety. While enjoyment of influencers' posts may reduce participant's risk perceptions of e-cigarettes, proactive creativity which centers on individual and collective efforts to reduce exposure to marketing appears to be beneficial and should be carefully examined in designing future interventions to reduce e-cigarette use. These interventions may include peer-led interactions on the best way of embedding anti-tobacco messaging into regular communications among young people. This may also include training potential influencers with an ability to raise awareness within the community and build public support for restrictions on e-cigarette marketing on social media.

## Data Availability

The raw data supporting the conclusions of this article will be made available by the authors, without undue reservation.

## References

[1] Kramarow EA Elgaddal N. Current Electronic Cigarette Use among Adults Aged 18 and Over: United States, 2021. NCHS Data Brief, No. 475. Hyattsville, MD: National Center for Health Statistics (2023). doi: 10.15620/cdc:129966 37486729

[2] Snell LM Barnes AJ Nicksic NE. A longitudinal analysis of nicotine dependence and transitions from dual use of cigarettes and electronic cigarettes: evidence from waves 1–3 of the PATH study. J Stud Alcohol Drugs. (2020) 81:595–603. doi: 10.15288/jsad.2020.81.595 33028472 PMC8076487

[3] National Center for Health Statistics. Percentage of Current Electronic Cigarette Use for Adults Aged 18 and Over, United States, 2019–2021. National Health Interview Survey (2021). Available online at: https://wwwn.cdc.gov/NHISDataQueryTool/SHS_adult/index.html (Accessed October 3, 2024).

[4] Wang JB Olgin JE Nah G Vittinghoff E Cataldo JK Pletcher MJ . Cigarette and e-cigarette dual use and risk of cardiopulmonary symptoms in the Health eHeart Study. PLoS ONE. (2018) 13:e0198681. doi: 10.1371/journal.pone.0198681 30044773 PMC6059385

[5] Culbreth RE Spears CA Brandenberger K Feresin R Self-Brown S Goodfellow LT . Dual use of electronic cigarettes and traditional cigarettes among adults: psychosocial correlates and associated respiratory symptoms. Respir Care. (2021) 66:951–9. doi: 10.4187/respcare.08381 33688088

[6] Bandi P Cahn Z Goding Sauer A Douglas CE Drope J Jemal A . Trends in e-cigarette use by age group and combustible cigarette smoking histories, U.S. adults, 2014–2018. Am J Prev Med. (2021) 60:151–8. doi: 10.1016/j.amepre.2020.07.026 33032869

[7] Obisesan OH Osei AD Uddin SMI Dzaye O Mirbolouk M Stokes A . Trends in e-cigarette use in adults in the United States, 2016–2018. JAMA Intern Med. (2020) 180:1394–8. doi: 10.1001/jamainternmed.2020.2817 32897288 PMC7489391

[8] Kalkhoran S Chang Y Rigotti NA. Electronic cigarette use and cigarette abstinence over 2 years among U.S. smokers in the Population Assessment of Tobacco and Health Study. Nicotine Tob Res. (2020) 22:728–33. doi: 10.1093/ntr/ntz114 31298296 PMC7171267

[9] Kalkhoran S Glantz SA. E-cigarettes and smoking cessation in real-world and clinical settings: a systematic review and meta-analysis. Lancet Respir Med. (2016) 4:116–28. doi: 10.1016/S2213-2600(15)00521-4 26776875 PMC4752870

[10] Chen R Pierce JP Leas EC White MM Kealey S Strong DR . Use of electronic cigarettes to aid long-term smoking cessation in the United States: prospective evidence from the PATH cohort study. Am J Epidemiol. (2020) 189:1529–37. doi: 10.1093/aje/kwaa161 32715314 PMC7705599

[11] Huang J Kornfield R Szczypka G Emery SL. A cross-sectional examination of marketing of electronic cigarettes on Twitter. Tob Control. (2014) 23:iii26–30. doi: 10.1136/tobaccocontrol-2014-051551 24935894 PMC4078681

[12] Shi R Khayat A Lee J Garrison KA Jebai R Wackowski OA . Electronic nicotine delivery system advertisement trends after US federal policy changes. JAMA Netw Open. (2025) 8:e2459188. doi: 10.1001/jamanetworkopen.2024.59188 39937476 PMC11822544

[13] US Food and Drug Administration. Vaporizers, E-cigarettes, and Other Electronic Nicotine Delivery Systems (ENDS) (2025). Available online at: http://www.fda.gov/tobaccoproducts/labeling/productsingredientscomponents/ucm45661 (Accessed July 21, 2025)

[14] Silver NA Bertrand A Kucherlapaty P Schillo BA. Examining influencer compliance with advertising regulations in branded vaping content on Instagram. Front Public Health. (2023) 10:1001115. doi: 10.3389/fpubh.2022.1001115 36699883 PMC9869128

[15] Vassey J Vogel EA Unger JB Cho J Bae D Donaldson SI . Impact of Instagram and TikTok influencer marketing on perceptions of e-cigarettes and perceptions of influencers in young adults: a randomised survey-based experiment. Tob Control. (2025). 35:358–65. doi: 10.1136/tc-2024-059021 PMC12343919 39947698

[16] Ribisl KM D'Angelo H Feld AL Schleicher NC Golden SD Luke DA . Disparities in tobacco marketing and product availability at the point of sale: results of a national study. Prev Med. (2017) 105:381–8. doi: 10.1016/j.ypmed.2017.04.010 28392252 PMC5630502

[17] Grilo G Crespi E Cohen JE. A scoping review on disparities in exposure to advertising for e-cigarettes and heated tobacco products and implications for advancing a health equity research agenda. Int J Equity Health. (2021) 20:238. doi: 10.1186/s12939-021-01576-2 34717629 PMC8557615

[18] Tan ASL Hanby EP Sanders-Jackson A Lee S Viswanath K Potter J. Inequities in tobacco advertising exposure among young adult sexual, racial and ethnic minorities: examining intersectionality of sexual orientation with race and ethnicity. Tob Control. (2021) 30:84–93. doi: 10.1136/tobaccocontrol-2019-055313 31857490

[19] Hackbarth DP Silvestri B Cosper W. Tobacco and alcohol billboards in 50 Chicago neighborhoods: market segmentation to sell dangerous products to the poor. J Public Health Policy. (1995) 16:213–30. doi: 10.2307/3342593 7560056

[20] Luke D Esmundo E Bloom Y. Smoke signs: patterns of tobacco billboard advertising in a metropolitan region. Tob Control. (2000) 9:16–23. doi: 10.1136/tc.9.1.16 10691754 PMC1748288

[21] Stoddard JL Johnson CA Sussman S Dent C Boley-Cruz T. Tailoring outdoor tobacco advertising to minorities in Los Angeles County. J Health Commun. (1998) 3:137–46. doi: 10.1080/108107398127427 10977250

[22] Primack BA Bost JE Land SR Fine MJ. Volume of tobacco advertising in African American markets: systematic review and meta-analysis. Public Health Rep. (2007) 122:607–15. doi: 10.1177/003335490712200508 17877308 PMC1936959

[23] Henriksen L Schleicher NC Dauphinee AL Fortmann SP. Targeted advertising, promotion, and price for menthol cigarettes in California high school neighborhoods. Nicotine Tob Res. (2012) 14:116–21. doi: 10.1093/ntr/ntr122 21705460 PMC3592564

[24] Toll BA Ling PM. The Virginia Slims identity crisis: an inside look at tobacco industry marketing to women. Tob Control. (2005) 14:172–80. doi: 10.1136/tc.2004.008953 15923467 PMC1748044

[25] Gibbons FX Fleischli ME Gerrard M Simons RL. Reports of perceived racial discrimination among African American children predict negative affect and smoking behavior in adulthood: a sensitive period hypothesis. Dev Psychopathol. (2018) 30:1629–47. doi: 10.1017/S0954579418001244 30451139

[26] Horton KD Loukas A. Discrimination, religious coping, and tobacco use among White, African American, and Mexican American vocational school students. J Relig Health. (2013) 52:169–83. doi: 10.1007/s10943-011-9462-z 21249522 PMC8117250

[27] Hicks MR Kogan SM. The influence of racial discrimination on smoking among young Black men: a prospective analysis. J Ethn Subst Abuse. (2020) 19:311–26. doi: 10.1080/15332640.2018.1511493 30372370 PMC6488444

[28] Rose SW Mayo A Ganz O Perreras L D'Silva J Cohn A. Perceived racial/ethnic discrimination, marketing, and substance use among young adults. J Ethn Subst Abuse. (2019) 18:558–77. doi: 10.1080/15332640.2018.1425949 29424638

[29] Wang TW Asman K Gentzke AS Cullen KA Holder-Hayes E Reyes-Guzman C . Tobacco product use among adults—United States, 2017. MMWR Morb Mortal Wkly Rep. (2018) 67:1225–32. doi: 10.15585/mmwr.mm6744a2 30408019 PMC6223953

[30] Kirchner TR Villanti AC Cantrell J Anesetti-Rothermel A Ganz O Conway KP . Tobacco retail outlet advertising practices and proximity to schools, parks and public housing affect Synar underage sales violations in Washington, DC. Tob Control. (2015) 24:e52–8. doi: 10.1136/tobaccocontrol-2013-051239 24570101

[31] Rose SW Anesetti-Rothermel A Westneat S van de Venne J Folger S Rahman B . Inequitable distribution of FTP marketing by neighborhood characteristics: further evidence for targeted marketing. Nicotine Tob Res. (2022) 24:484–92. doi: 10.1093/ntr/ntab222 34687204 PMC8887586

[32] Dauphinee AL Doxey JR Schleicher NC Fortmann SP Henriksen L. Racial differences in cigarette brand recognition and impact on youth smoking. BMC Public Health. (2013) 13:170. doi: 10.1186/1471-2458-13-170 23442215 PMC3586353

[33] Rose SW Anesetti-Rothermel A Elmasry H Niaura R. Young adult non-smokers' exposure to real-world tobacco marketing: results of an ecological momentary assessment pilot study. BMC Res Notes. (2017) 10:1–6. doi: 10.1186/s13104-017-2758-7 28859667 PMC5580291

[34] Sells JR Lammers JP Okuyemi KS Izmirlian G. Pro-tobacco advertisement exposure among African American smokers: an ecological momentary assessment study. Addict Behav. (2018) 83:142–7. doi: 10.1016/j.addbeh.2017.10.015 29174665 PMC5916503

[35] Chen-Sankey JC Unger JB Bansal-Travers M Niederdeppe J Bernat E Choi K. E-cigarette marketing exposure and subsequent experimentation among youth and young adults. Pediatrics. (2019) 144:e20191119. doi: 10.1542/peds.2019-1119 31659003 PMC6836725

[36] Pettigrew S Santos JA Pinho-Gomes AC Li Y Jones A. Exposure to e-cigarette advertising and young people's use of e-cigarettes: a four-country study. Tob Induc Dis. (2023) 21:141. doi: 10.18332/tid/172414 37881174 PMC10594952

[37] Pettigrew S Santos JA Li Y Jun M Anderson C Jones A. Short report: factors contributing to young people's susceptibility to e-cigarettes in four countries. Drug Alcohol Depend. (2023) 250:109944. doi: 10.1016/j.drugalcdep.2023.109944 37316389

[38] Donaldson SI Dormanesh A Perez C Majmundar A Allem JP. Association between exposure to tobacco content on social media and tobacco use: a systematic review and meta-analysis. JAMA Pediatr. (2022) 176:878–85. doi: 10.1001/jamapediatrics.2022.2223 35816331 PMC9274450

[39] Vogel E Barrington-Trimis J Vassey J Soto D Unger J. Young adults' exposure to and engagement with tobacco-related social media content and subsequent tobacco use. Nicotine Tob Res. (2024) 26:S3–12. doi: 10.1093/ntr/ntad108 38366337 PMC10873498

[40] Kheirallah KA Cobb CO Alsulaiman JW Alzoubi A Hoetger C Kliewer W . Trauma exposure, mental health and tobacco use among vulnerable Syrian refugee youth in Jordan. J Public Health. (2020) 42:e343–51. doi: 10.1093/pubmed/fdz128 31742341

[41] Kozaric-Kovacic D Ljubin T Grappe M. Comorbidity of posttraumatic stress disorder and alcohol dependence in displaced persons. Croat Med J. (2000) 41:173–8. Available online at: https://www.cmj.hr/2000/41/2/10853047.pdf 10853047

[42] Giuliani KK Mire O Leinberger-Jabari A Ehrlich LC Stigler MH Pryce DJ . Cigarettes and the Somali diaspora: tobacco use among Somali adults in Minnesota. Am J Prev Med. (2012) 43:S205–13. doi: 10.1016/j.amepre.2012.08.002 23079218

[43] Wilhelm AK Parks MJ Eisenberg ME Allen ML. Patterns of tobacco use and related protective factors among Somali youth in the United States. J Immigr Minor Health. (2021) 23:103–12. doi: 10.1007/s10903-020-01013-6 32333287

[44] Fife ST Gossner JD. Deductive qualitative analysis: evaluating, expanding, and refining theory. Int J Qual Methods. (2024) 23. doi: 10.1177/16094069241244856

[45] Kessler RC Andrews G Colpe LJ Hiripi E Mroczek DK Normand SL . Short screening scales to monitor population prevalences and trends in non-specific psychological distress. Psychol Med. (2002) 32:959–76. doi: 10.1017/S0033291702006074 12214795

[46] Braun V Clarke V. Using thematic analysis in psychology. Qual Res Psychol. (2006) 3:77–101. doi: 10.1191/1478088706qp063oa

[47] MacMonegle A Bennett M Speer JL O'Brien EK Pitzer L Jaarsma A . Evaluating the real cost digital and social media campaign: longitudinal effects of campaign exposure on e-cigarette beliefs. Nicotine Tob Res. (2024) 26:S19–26. doi: 10.1093/ntr/ntad185 38366338

[48] Lyu J Meacham M Nguyen N Ramo D Ling P. Factors associated with abstinence among young adult smokers enrolled in a real-world social media smoking cessation program. Nicotine Tob Res. (2024) 26:S27–35. doi: 10.1093/ntr/ntad170 38366340 PMC10873491

[49] Lovato C Watts A Stead LF. Impact of tobacco advertising and promotion on increasing adolescent smoking behaviours. Cochrane Database Syst Rev. (2011) 2011:CD003439. doi: 10.1002/14651858.CD003439.pub2 21975739 PMC7173757

[50] Jenssen BP Klein JD Salazar LF Daluga NA DiClemente RJ. Exposure to tobacco on the internet: content analysis of adolescents' internet use. Pediatrics. (2009) 124:e180–6. doi: 10.1542/peds.2008-3838 19620193 PMC2818533

[51] Robbins BW McLaughlin S Finn PW Spencer AL Coleman DL. Young adults: addressing the health needs of a vulnerable population. Am J Med. (2020) 133:999–1002. doi: 10.1016/j.amjmed.2020.04.005 32387083

[52] Mohamed AA Lantz K Ahmed YA Osman A Nur MA Nur O . An assessment of health priorities among a community sample of Somali adults. J Immigr Minor Health. (2022) 24:455–60. doi: 10.1007/s10903-021-01166-y 33740189 PMC7975235

[53] Scuglik DL Alarcon RD. Growing up whole: Somali children and adolescents in America. Psychiatry. (2005) 2:20–31. PMC3000212 21152169

[54] Invitto S Moselli P. Exploring embodied and bioenergetic approaches in trauma therapy: observing somatic experience and olfactory memory. Brain Sci. (2024) 14:385. doi: 10.3390/brainsci14040385 38672034 PMC11048503

[55] Youssef NA. Potential societal and cultural implications of transgenerational epigenetic methylation of trauma and PTSD: pathology or resilience? Yale J Biol Med. (2022) 95:171–4. 35370497 PMC8961703

[56] Kong J Lou C. Beyond persuasion knowledge: examining the roles of visual appeal, visual congruence, and social proof in influencer advertising. J Retail Consum Serv. (2026) 88:104502. doi: 10.1016/j.jretconser.2025.104502

[57] Chen-Sankey J Weiger C La Capria K Vassey J Jeong M Phan L . Young adults' visual attention to features of social media marketing for disposable e-cigarettes and associated perceptions. Addiction. (2025) 120:439–51. doi: 10.1111/add.16586 38923723 PMC11813734

[58] Opoku D Donkor C Yeboah JNO Quagraine L. Navigating the relationship between social media use and mental health in the digital age. Discover Mental Health. (2025) 5:149. doi: 10.1007/s44192-025-00285-4 41057730 PMC12504152

[59] Hastings G Stead M Webb J. Fear appeals in social marketing: strategic and ethical reasons for concern. Psychol Mark. (2004) 21:961–86. doi: 10.1002/mar.20043

[60] Tandoc EC Ferrucci P Duffy M. Facebook use, envy, and depression among college students: is facebooking depressing? Comput Hum Behav. (2015) 43:139–46. doi: 10.1016/j.chb.2014.10.053

[61] Weeks BE. Emotions, partisanship, and misperceptions: how anger and anxiety moderate the effect of partisan bias on susceptibility to political misinformation. J Commun. (2015) 65:699–719. doi: 10.1111/jcom.12164

[62] Metzler H Garcia D. Social drivers and algorithmic mechanisms on digital media. Perspect Psychol Sci. (2024) 19:735–48. doi: 10.1177/17456916231185057 37466493 PMC11373151

[63] Pellegrino A Abe M Shannon R. The dark side of social media: content effects on the relationship between materialism and consumption behaviors. Front Psychol. (2022) 13:870614. doi: 10.3389/fpsyg.2022.870614 35572231 PMC9096894

[64] Liu D He B Feng R Huang X Liu G. How social media sharing drives consumption intention: the role of social media envy and social comparison orientation. BMC Psychol. (2024) 12:157. doi: 10.1186/s40359-024-01627-7 38491525 PMC10943867

[65] Wang Y Xu J Lu Y. Associations among trauma exposure, post-traumatic stress disorder, and depression symptoms in adolescent survivors of the 2013 Lushan earthquake. J Affect Disord. (2020) 264:407–13. doi: 10.1016/j.jad.2019.11.067 31791678

[66] Booth P Albery IP Frings D. Effect of e-cigarette advertisements and antismoking messages on explicit and implicit attitudes towards tobacco and e-cigarette smoking in 18-65-year-olds: a randomised controlled study protocol. BMJ Open. (2017) 7:e014361. doi: 10.1136/bmjopen-2016-014361 28645957 PMC5623370

[67] Tan CY Chuah CQ Lee ST Tan CS. Being creative makes you happier: the positive effect of creativity on subjective well-being. Int J Environ Res Public Health. (2021) 18:7244. doi: 10.3390/ijerph18147244 34299693 PMC8305859

[68] Zhao R Tang Z Lu F Xing Q Shen W. An updated evaluation of the dichotomous link between creativity and mental health. Front Psychiatry. (2022) 12:781961. doi: 10.3389/fpsyt.2021.781961 35111087 PMC8802834

