# Supplementary material for: Perceptions of Somali American male young adults in Salt Lake City about social media marketing of e-cigarettes: a pilot study
Source: Front Public Health. 2026 Jul 15;14:1819564. doi: 10.3389/fpubh.2026.1819564 (PMC13414751; doi:10.3389/fpubh.2026.1819564)

Supplementary Material

Supplementary Table 1: Focus Group Discussion Guide

1. Experiences with vaping and social media.

Question: Can you describe your general experience with vaping and social media?

1. Experiences with targeted marketing on social media.
2. What is your experience with getting unwanted messages on social media.
3. How do you feel when you get more e-cigarette messages on social media compared to your peers?
4. Experiences with social media marketing of e-cigarettes.

Transition - Think back to when you first experienced vaping advertisements on social media. What was your first impression?"

Key questions:

1. What is your general perception about targeted marketing of e-cigarettes?

Probing questions:

1. Can you describe to us how you concluded that you were being targeted
2. Can you please expand on these experiences?
3. Can you describe the kind or messages/promotions and advertisement that you get from influencers on social media.
4. Is there anything that you do to reduce e-cigarette advertisements that you see on social media?
5. To understand the psychological effects of targeted marketing of e-cigarettes.

Question: Please describe your specific experiences with targeted marketing of e-cigarettes.

Exploratory question: Describe your experience if you have ever felt any of the emotions described below:

i. Nervous, anxious, or on edge.

ii. Sad or depressed.

iii. Hopeless.

iv. Everything was an effort.

v. Worthless.

vi. Restless or fidgety.

Exploratory question: Can you explain why you felt this way?

1. To understand your perception of how your culture or race may have made you a target for e-cigarette product marketing.

Questions:

i. Why do you think you are/were targeted? Is there any link to your faith/race or culture?

ii. Can you describe how others socially perceive you if they see cigarette messages on your phone or device?

Supplementary Table 2: Themes, sub-themes and additional illustrative quotes

| Themes | Sub-themes | Illustrative Quotes |
| --- | --- | --- |
| Intrusiveness | Opaque algorithms | - *“I receive e-cigarette messages whenever I chat with others about pods.” (Participant in FGD 1)* - *“I’ve learned not to view vaping messages. When you do, they keep coming.” (Participant in FGD 1)* - *“I have bought vaping products online in the past. Now I get both e-cigarette and vaping cessation messages.” (Participant in FGD 3)* - *“I recall searching for some vaping products out of curiosity in the past but have continued to get e-cigarette promotion prompts.” (Participant in FGD 2)* - *“I feel uncomfortable when opening my social media handles after discussion with friends about cigarettes or e-cigarettes because the advertisements start coming in” (Participant in FGD 1)* |
|  | Trauma amplification | - *“Every time I see an e-cigarette advert, I feel guilty because I used to vape in middle school.” (Participant in FGD 2)* - *“These vaping messages remind me of a bad experience I had when I was caught smoking in middle school. The shame was overwhelming.” (Participant in FGD 2)* - *“I don’t want to be labelled as a cigarette smoker again. I had that label all through high school because older people don’t seem to know the difference between tobacco products.’ (Participant in FGD 3)* - *“In Islam, cigarettes are impure substances which should never be put in the mouth. I feel embarrassed when my Muslim friends see vaping advertisements on my phone because they know that I used to smoke.” (Participant in FGD 3)* |
|  | Manipulation of social media pages and ‘click baiting’ | - *“I have received many messages that I did not know were related to vaping until I clicked the link.” (Participant in FGD 2)* - *“I get prompted to view pens without knowing that they are not writing pens.” (Participant in FGD 2)* - *“I am wary about visiting sites that sell disposable e-cigarette paraphernalia because I continue to get the messages afterwards.” (Participant in FGD 3)* |
|  | Manipulation of information | - *“I get a lot of e-cigarette content from young, Black influencers.” (Participant in FGD 2)* - *“I get prompted to view messages from people about my age who are promoting e-cigarettes. Most of my friends have received similar messages.” (Participant in FGD 1)* - *“I get pictures of pods that look like common fruits.” (Participant in FGD 1)* |
| Vulnerability | Feelings of insecurity due to unrealistic expectations | - *“All the messages I get are from well-dressed people my age and I always feel like I need to do more about my dressing.” (Participant in FGD 1)* - *“The background of vaping pictures always looks colorful and cool. They usually have a pen in one hand and a symbol of success, for example, a good-looking car on the other.” (Participant in FGD 1)* - *“The people in the videos look happy and remind me of the need to be successful.” (Participant in FGD 2)* - *“I love watching the videos and I can’t stop watching them despite the fact that they make me sad afterwards.” (Participant in FGD 2)* |
|  | Feeling of inadequacy due to inability to predict outcomes | - *“I have bought vaping products online in the past. Now I get both e-cigarette and vaping cessation messages.” (Participant in FGD 3)* - *“I recall searching for some products out of curiosity in the past but have continued to get e-cigarette promotion prompts.” (Participant in FGD 1)* - *“I feel vulnerable when I open my social media handles especially when my friends are around because I do not know what to expect” Participant in FGD 1)* |
|  | Inability to keep social media engagement focused | - *“I’ll continue to indicate my lack of interest in e-cigarette materials. I am interested in religious content and look forward to a time when only religious content will get to me.” (Participant in FGD 3)* - *“I create new accounts and then stop using them after some time. I feel like there is very little choice that I have about content.” (Participant in FGD 2)* |
| Anxiety | Uncertainty about messages and interaction on social media | - *“I report vaping advertisement that ‘pop up’ on my page. However, the messages continue for such time that I believe I need to do more with my life.” (Participant in FGD 1)* - *“The vaping advertisements on my phone come up at the wrong time.” (Participant in FGD 3)* - *“Vaping advertisements do not only appear on my phone. They appear on all devices linked to me including my email.” (Participant in FGD 3)* |
|  | Negative reaction from peers (especially females) | - *“I always fear that my female friends will see the e-cigarette adverts and would be disappointed in me.”- (Participant in FGD 1* - *“I feel uncomfortable anytime I open my browser when my female friends are nearby.” (Participant in FGD 1)* - *“The vaping advertisements on my phone pop up at the wrong time.” (Participant in FGD 3)* |
|  | Emotional frustration from power imbalance in interaction with social media companies | - *“Social media companies make much money from the adverts. I don’t think they are interested in the effects of the e-cigarette advertisements.” (Participant in FGD 3)* - *“The report features function on the sites are not usually responsive. Yes, I notice some reduction in the number of e-cigarette messages after I activate the function, but that happens much later.” (Participant in FGD 2)* - *“I immediately report unwanted e-cigarette advertisement using the built-in help center in Instagram. However, it takes a lot of time before it goes away. I don’t know what else I can do to make it go away faster.” (Participant in FGD2)* |
| Enjoyment | Enjoyment of images | - *“I feel like I’m not prepared for more social media use. I use little social media these days. There is so much to enjoy looking at in the nice clothes and good-looking people.” (Participant in FGD 2)* - *“I agree with people who say that there is more to social media than what we see. However, I like what I see, and I enjoy watching the people.” (Participant in FGD 3)* |
|  | Enjoyment of interaction with influencers | - *“I enjoy interacting and following the influencers (e-cigarette influencers) on social media because they are the leaders and I could benefit if they follow me back.” (Participant in FGD 1)* - *“The influencers who talk about vaping are very approachable and real. They look happy and remind me of the need to be successful.” (Participant in FGD 2)* - *“I see the things (paraphernalia) used to smoke e-cigarettes every time online. I can easily recognize them when I see them with young people.” (Participant in FGD 2)* |
| Pro-active creativity | Creativity at individual level | - *“I have had some peace since I adjusted my social media privacy settings which makes it difficult for marketers to target my account.” (Participant in FGD 1)* - *“I have learnt to only buy (e-cigarette) cartridges and pods with cash in the shops and gas stations.” (Participant in FGD 2)* - *“I deliberately delete tracking identifiers on my computer to block feedback to advertising (e-cigarette advertising) sites. I have found that this helps me a lot.” (Participant in FGD 2)* |
|  | Creativity at group level | - *“When I see e-cigarette adverts on my friends’ phones, I muster the courage to start the conversation by telling them to look out for a report link or button on their App.” (Participant in FGD 2)* - *“I know that e-cigarette targeted messages wouldn’t stop until we do something about it. I have shared information with my friends about how they can get the messages to stop.” (Participant in FGD 1)* |


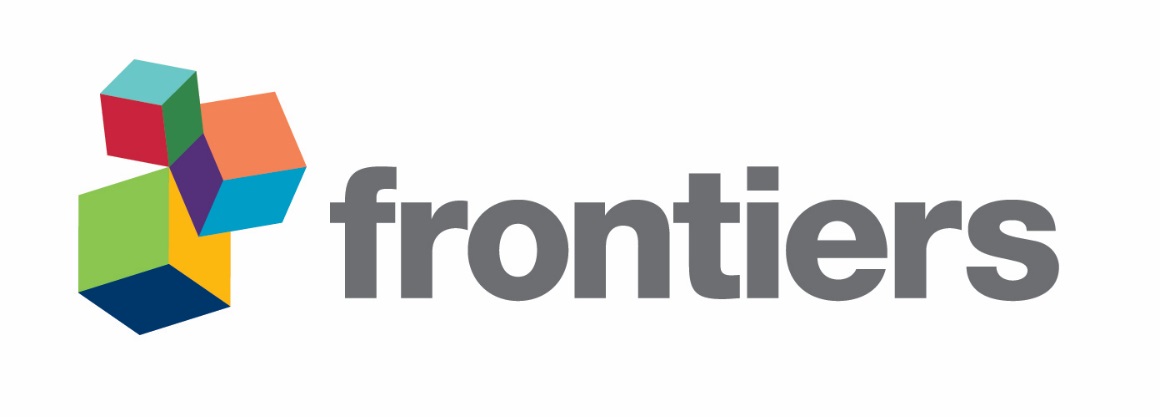

Supplement: Supplementary file 1 [file Data_Sheet_1.docx]
